# Comparative Safety of Dipeptidyl Peptidase-4 Inhibitors Versus Sulfonylureas and Other Glucose-lowering Therapies for Three Acute Outcomes

**DOI:** 10.1038/s41598-018-33483-y

**Published:** 2018-10-11

**Authors:** John-Michael Gamble, Jennifer R. Donnan, Eugene Chibrikov, Laurie K. Twells, William K. Midodzi, Sumit R. Majumdar

**Affiliations:** 1 School of Pharmacy, Faculty of Science, University of Waterloo, 10A Victoria Street South, Kitchener, Ontario, N2G 2C5 Canada; 2 School of Pharmacy, Memorial University of Newfoundland, 300 Prince Philip Drive, St. John’s, Newfoundland and Labrador, A1B 3V6 Canada; 3 Faculty of Medicine, Memorial University of Newfoundland, 300 Prince Philip Drive, St. John’s, Newfoundland and Labrador, A1B 3V6 Canada; 4 Division of General Internal Medicine, Department of Medicine, University of Alberta, Edmonton, Alberta T6G 2B7 Canada

## Abstract

Although the glucose lowering effect of dipeptidyl peptidase-4 (DPP4) inhibitors is well established, several potential serious acute safety concerns have been raised including acute kidney injury, respiratory tract infections, and acute pancreatitis. Using the UK-based Clinical Practice Research Datalink (CPRD), we identified initiators (365-day washout period) of DPP4 inhibitors and relevant comparators including initiators of sulfonylureas, metformin, thiazolidinediones, and insulin between January 2007 and January 2016 to quantify the association between DPP4 inhibitors and three acute health events – acute kidney injury, respiratory tract infections, and acute pancreatitis. The associations between drug and study outcomes were estimated using Cox proportional hazard models adjusted for deciles of high-dimensional propensity scores and number of additional glucose lowering agents. After controlling for potential confounders, the risk was not significantly increased or decreased for initiators of DPP4 inhibitors compared to sulfonylureas (hazard ratio (HR) [95% confidence interval (CI)] for acute kidney injury: 0.81 [0.56–1.18]; HR for respiratory tract infections: 0.93 [0.84–1.04]; HR for acute pancreatitis 1.03 [0.42–2.52], metformin (HR for respiratory tract infection 0.91 [0.65–1.27]), thiazolidinediones (HR for acute kidney injury: 1.12 [0.60–2.10]; HR for respiratory tract infections: 1.02 [0.86–1.21]; HR for acute pancreatitis: 1.21 [0.25–5.72]), or insulin (HR for acute kidney injury: 1.40 [0.77–2.55]; HR for respiratory tract infections: 0.74 [0.60–0.92]; HR for acute pancreatitis: 1.01 [0.24–4.19]). Initiators of DPP4 inhibitors were associated with an increased risk of acute kidney injury when compared to metformin initiators (HR [95% CI] for acute kidney injury: 1.85 [1.10–3.12], although this association was attenuated when DPP4 inhibitor monotherapy was compared to metformin monotherapy exposure as a time-dependent variable (HR 1.39 [0.91–2.11]). Initiation of a DPP4 inhibitor was not associated with an increased risk of acute kidney injury, respiratory tract infections, or acute pancreatitis compared to sulfonylureas or other glucose-lowering therapies.

## Introduction

The glucose-lowering effects of dipeptidyl peptidase-4 (DPP4) inhibitors have been well documented since their introduction to the global market in the mid-2000’s. Their utilization for the management of glycemic control in patients with type 2 diabetes is increasing^1–3^. Despite beneficial glycemic effects, a low risk of hypoglycemia, and neutral effect on weight, there is a lack of evidence suggesting any mortality or morbidity benefits for patients using DPP4 inhibitors^4,5^. Moreover, several potential acute effects of DPP4 inhibitors have been generated from pre-marketing and post-marketing data including clinical trials, pharmacovigilance databases, and observational studies. These include health events such as acute kidney injury, respiratory tract infections, and acute pancreatitis.

It is unclear whether DPP4 inhibitors play a role in the development of diabetic kidney disease. DPP4 inhibitors prolong the half-life of glucagon-like-peptide-1 (GLP-1), which in turn improves insulin secretion in response to oral glucose consumption and suppresses glucagon release, ultimately decreasing blood glucose levels. Given that the DPP4 enzyme is present in various components of the endothelial and epithelial kidney tissues (including renal proximal tubular epithelia, podocytes, mesangial cells, and pre-glomerular vascular smooth muscle cells), it has been hypothesized that DPP4 inhibitors will have a protective effect on the kidney by reducing inflammation and fibrosis and improving overall function^6,7^. However, other mechanisms may be responsible for acute changes in renal function including fluid depletion and volume contraction via vomiting and diarrhea, although evidence exists suggesting a beneficial effect of natriuretic and diuretic properties of DPP4 inhibitors^8,9^. Findings from observational studies have been inconsistent. A nested case-control study of over 7000 patients in Taiwan found that individuals who had taken a DPP4 inhibitor in the last 365 days were more likely to develop acute kidney injury (OR = 1.2; 95% CI 1.11–1.37)^10^. Sub-group analysis showed this increased risk was primarily in individuals who had taken a DPP4 inhibitor in the last 30 days. A recent cohort study, also using a Taiwanese database, included 923,936 patients with diabetes, 83,638 of which were users of a DPP4 inhibitor. After an average of 3.6 years of follow-up, DPP4 inhibitors users had a significantly lower risk of acute kidney injury (HR = 0.57; 95% CI 0.53–0.61) and acute kidney injury requiring dialysis (HR = 0.57; 95% CI 0.49–0.66)^11^. Another cohort study using administrative data sources in the United Kingdom and the United States, included 1,024,124 individuals, 110,740 exposed to the DPP-4 inhibitor saxagliptin. With follow-up time ranging from 5.6–8.1 months, this study found no increased risk of acute kidney injury (HR = 0.99; 95% CI 0.88–1.11)^12^.

There are several potential mechanisms that may be responsible for immune-related effects of DPP4 inhibitors. Biologically, DPP4 has immune modulatory effects on cell growth, differentiation, apoptosis, and inflammatory cytokines^13^. Since the enzyme DPP4 is structurally similar to the lymphocyte protein CD26, there is a concern that DPP4 inhibitors may increase the risk of infections^14,15^. Spontaneous reporting of infections are two times higher in patients using DPP4 inhibitors compared to metformin, with reports of upper respiratory tract infections at 12 times higher^13^; however, there are significant limitations with spontaneous reporting and, due to underreporting of events, population-based incidence rates cannot be measured^16^. Results from pre-marketing clinical trials of DPP4 inhibitors are inconsistent with some drugs showing a potential increased dose-dependent risk of upper respiratory tract infections (e.g., sitagliptin 100 mg 11.4%, 200 mg 14.8%, placebo 7.1%) and other drugs showing no increased risk (e.g., saxagliptin)^15^. A recently published meta-analysis pooling these trials did not find a difference in the rate of respiratory tract infections between users of DPP4 inhibitors and other agents^17^; however, the included trials were of short duration and not designed to assess long-term safety. Recent observational studies have also failed to show any relationship between DPP4 inhibitors and community-acquired pneumonia^18,19^.

Concerns over an increased risk of acute pancreatitis with the DPP4 inhibitors and GLP-1 receptor agonists have been raised based on case reports^20–22^. Some animal and post-mortem human studies suggest that activation of GLP-1 receptor on exocrine pancreatic cells can lead to their proliferation and possibly to inflammation^23–25^. However, a causal association between exposure to incretin-based medications and pancreatitis has not been established and both the US Food and Drug Administration (FDA) and European Medicines Association (EMA) have investigated the association thoroughly^20,24,26^. There is emerging evidence that ductal and pancreatic stellate cells of chronically inflamed pancreas express GLP-1 receptors that are not normally present in the cells of healthy pancreas^27^. This observation makes it possible for patients with pre-existing undiagnosed asymptomatic chronic pancreatitis to experience acute episodes after initiation of GLP-1 agonist or DPP4 inhibitor contributing to the increased incidence of acute pancreatitis in some studies. The findings from the majority of published observational studies (8 of 10) suggest that incretin-based medications do not increase the risk of acute pancreatitis, although the evidence is not unanimous^28–37^.

It is important that we continue to investigate the risk of serious acute events with these agents, especially given the susceptibility to impaired kidney function, infection^38,39^, and acute pancreatitis. Furthermore, there is limited evidence evaluating the association between DPP4 inhibitors and acute outcomes, notably acute kidney injury and respiratory tract infections. Therefore, we conducted a series of population-based cohort studies to estimate the association between DPP4 inhibitors and these important acute outcomes.

## Methods

### Study Design and Data Sources

The Clinical Practice Research Datalink (CPRD) GOLD database was used to conduct a cohort study to estimate the risks of acute kidney injury, respiratory tract infections and acute pancreatitis in new users of DPP4 inhibitors compared to new users of glucose-lowering therapies with type 2 diabetes. The CPRD GOLD database contains longitudinal information from over 650 general practitioner practice sites across the United Kingdom, equating to about 7% of the total population and has been shown to be representative of the broader UK population^40^. It also contains a wide variety of patient information including sociodemographic data, physiological measures, laboratory data, clinician-assigned diagnoses, and outpatient prescription records. Data from the CPRD is subject to a rigorous quality check prior to being released for research purposes^40^. The source population for this study was derived based on the February 2016 CPRD GOLD dataset build. Furthermore, a subgroup of our source population (~58%) was linked to 3 additional databases: (a) Hospital Episode Statistics (HES – data available up to March 31, 2014) providing diagnostic and clinical information for hospital visits, (b) the Office of National Statistics (ONS – data available up to April 30, 2014) providing cause of death, and (c) the index of multiple deprivation (2010) providing an indicator for socioeconomic status.

The study population consisted of individuals whom 1) had filled a prescription for a glucose-lowering drug or had a diagnostic record for type 2 diabetes on or after January 1st, 2007 (index date), with no previous glucose-lowering prescription or diagnostic record within the previous 365 days (date of prescription/diagnostic record was set at the study entry date); 2) had up-to-standard medical history for a minimum of 12 months prior to study entry date; and 3) were 18 years of age or older at the study entry date. Women with polycystic ovarian syndrome, pregnant women, and those with gestational diabetes were excluded. A series of study cohorts were established to examine the three primary outcomes of interest (herein referred to as the acute kidney injury cohort, respiratory tract infection cohort, and acute pancreatitis cohort). We applied specific exclusion criteria based on pre-existing comorbidities, procedures, and prescription records (Supplemental material, Table S1). Moreover, separate study sub-cohorts were constructed for each exposure contrast of interest.

The study protocol was approved by the Independent Scientific Advisory Committee (ISAC 15_016RARA, August 2017) and the Health Research Ethics Board at Memorial University (HREB #20140717).

### Outcome and Exposure Definitions

Our primary outcomes of interest were time to the first diagnosis of either acute kidney injury, acute respiratory tract infection, or acute pancreatitis recorded during the study follow-up period in the respective cohort populations. Secondary outcomes included a breakdown of the types of acute respiratory infections (i.e., upper respiratory tract infection, pneumonia and influenza) within the respiratory tract infections cohort. Outcomes were defined based on READ codes contained in the CPRD data and ICD-10 codes in the HES data (see Supplementary Appendix Tables S2–S7 for list of diagnostic codes).

We defined exposure status in a consistent manner across all study sub-cohorts of interest. Sub-cohorts of interest were constructed based on exposure contrasts of interest that were defined a priori including a) DPP4 inhibitor vs. sulfonylurea, b) DPP4 inhibitor vs. metformin, c) DPP4 inhibitor vs. thiazolidinedione, d) DPP 4 inhibitor vs. insulin. Exposure status was defined as initiation (based off first time use with a 365-day washout period) of one of the following medication classes: 1) DPP4 inhibitors, 2) Glucagon-like peptide-1 (GLP-1) receptor agonists, 3) Sulfonylureas, 4) Metformin, 5) Thiazolidinediones, 6) Sodium glucose co-transptor-2 (SGLT2) inhibitors, 7) Meglitinides, 8) Acarbose, 9) Insulin, 10) diet/lifestyle management (no anti-diabetic medications). Person-time was accumulated in each of the exposure categories starting on the sub-cohort entry date. Censoring occurred once they discontinued the medication, received a prescription for a comparator medication, left the CPRD participating practice, died or on the last day of documented follow-up, whichever occurred first. To account for potential non-adherence, we applied 50% of the supplied number of days to the end of each prescription.

### Statistical Analysis

New initiators of DPP4 inhibitors were compared against sulfonylureas, metformin, thiazolidinediones, and insulin as active comparators within separate sub-cohorts. Sulfonylureas were pre-specified as our primary reference group and other glucose-lowering agents (e.g., SGLT-2 inhibitors) were used too infrequently to conduct any meaningful analysis. Multivariable Cox Proportional Hazards regression analysis was used to estimate the independent association between use of a DPP4 inhibitor and the risk of acute kidney injury, respiratory tract infections and acute pancreatitis, after adjusting for several potential confounding variables. We used high-dimensional propensity scores (hdPS) to adjust for potential confounding whereby we selected a set of 40 covariates, for each of the outcomes, from hundreds of potential confounders through an empirical, multi-step process^41^. Propensity scores for DPP4 inhibitors and comparators (separate models were run for each exposure contrast of interest) were estimated using multivariable logistic regression, whereby 40 covariates were identified through the hdPS procedure and several pre-defined covariates measured within the 365 days prior to the index date. Pre-defined covariates (Supplementary Appendix Table S8) included age, sex, smoking status, socioeconomic status, year of cohort entry, alcohol abuse, body mass index, duration of diabetes, history of cirrhosis, heart failure, hypertension, hyperlipidemia, ischemic heart disease, peripheral vascular disease, number of hospitalizations, number of distinct prescription drugs, most recent HbA1c value, as well as outcome specific covariates (e.g. prior use of ACE inhibitors for acute kidney injury; prior use of antibiotics for respiratory tract infection; and prior use of fibrates for acute pancreatitis). Covariates included in the final Cox Proportional Hazards model included deciles of the propensity scores, as well as a categorical variable indicating the number of glucose-lowering agents an individual was exposed to during follow-up (1, 2, or 3+). Models assumptions (i.e. proportional hazards assumption) were tested using standard diagnostics based on weighted residuals^42^.Our power calculation was based on the method of Schoenfeld, which is designed for censored outcome data. Power calculations were conducted a priori using Stata/MT 13.1 (command stpower cox) with a type 1 error rate of 5% and used feasibility counts from CPRD for number of exposed and unexposed patients. Assuming a 0.1% event rate^28^ [expected rate of acute pancreatitis which was the least frequent outcome] over a median of 1-year follow-up there was 72% power to detect a hazard ratio (HR) of 0.8 or smaller or 1.2 and greater.

We conducted additional subgroup and sensitivity analyses. First, we repeated the main analysis for acute kidney injury using a sub-group of patients with in-hospital events only. Second, a sub-group analysis based on location of respiratory tract infection was conducted including upper respiratory tract and lower respiratory tract (pneumonia/influenza). Third, we controlled for confounding by repeating the main analysis using a cohort of patients that were matched 1:1 based on their propensity score. A greedy nearest neighbor approach was used, and patients were selected in random order with a matching caliper set to 0.2 times the standard deviation of the natural logarithm of the propensity score. Fourth, we repeated the main analysis using a restricted cohort of patients that could be linked to hospitalization records. Fifth, we repeated our main analysis using alternative definitions of drug exposure including restricting to monotherapy users, add-on to metformin monotherapy users, and categorizing DPP4 inhibitors and comparators as time-dependent variables throughout follow-up. Lastly, we conducted an analysis whereby patients were grouped with others who had identical ordering of exposure to other glucose-lowering medication classes^43^. All analyses were conducted with R version 3.3.3.

## Results

**Figure 1:**
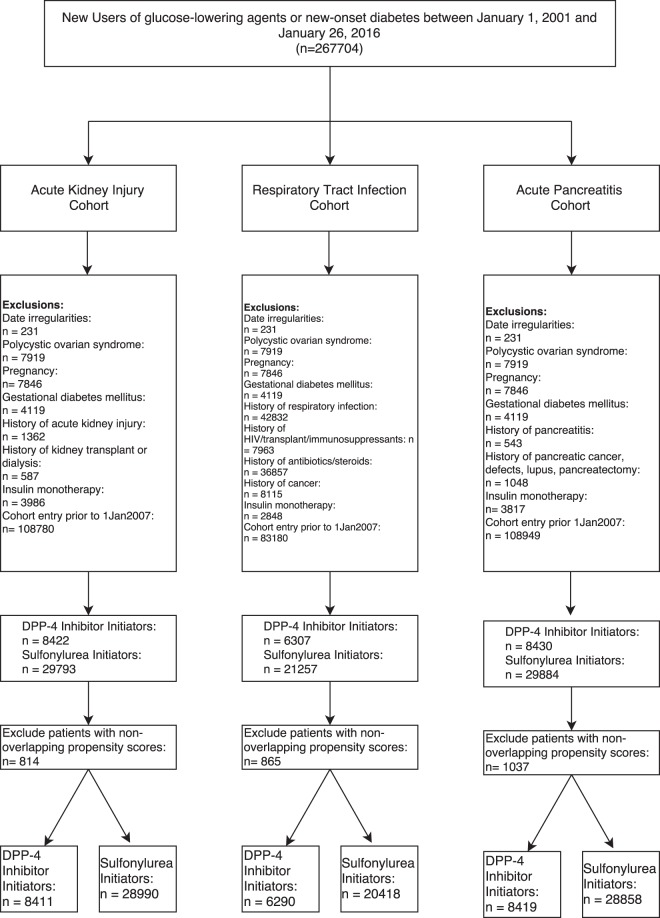
Flow diagram to identify new-users of DPP4 inhibitors and sulfonylureas for each study cohort.

A total of 267,704 patients were included in our source population (Fig. 1). Of these, 139,285 were eligible for the acute kidney cohort, 103,159 were eligible for the acute respiratory tract cohort, and 139,518 were eligible for the acute pancreatitis cohort.

**Figure 2:**
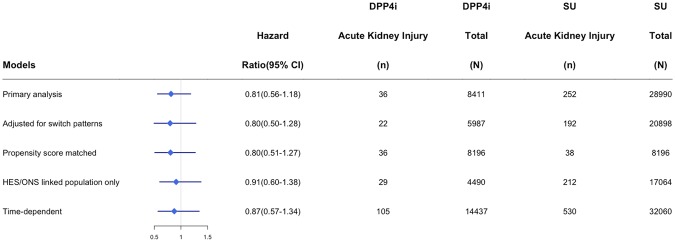
Sensitivity analyses for the association between DPP4 Inhibitors (DPP4i) and Sulfonylurea (SU) users for acute kidney injury.

Within the acute kidney injury cohort, there were 8,411 DPP4 inhibitor and 28990 sulfonylurea initiators. Mean follow-up time was 307 days and ranged from 1 to 3300 days. On average, DPP4 inhibitor users had diabetes for a longer duration (2.0 years vs. 1.0 years), had few hospitalizations, were less likely to have a HbA1C above 9%, and were more likely to have been on metformin in the year prior to cohort entry (Table 1). However, after propensity score matching, baseline differences between groups were comparable (Supplementary Appendix Table S9). Within 31,470 years of person-time follow-up this cohort experienced 288 episodes of acute kidney injury among new users of DPP4 inhibitors (n = 36, incidence rate = 4.8 per 1000 person-years) and sulfonylureas (n = 252, incidence rate 10.5 per 1000 person-years) (Table 2). Unadjusted hazard ratios (HR) suggest a decreased risk of acute kidney injury among DPP4 inhibitors users compared to sulfonylurea users (HR = 0.46; 95% CI 0.32–0.65); however, following adjustment for potential confounding variables, the association attenuates to a neutral one (adjusted HR = 0.81; 95% CI 0.56–1.18). No significant associations were observed comparing DPP4 inhibitor initiators with thiazolidinedione initiators (adjusted HR = 1.12; 95% CI 0.60–2.10) or insulin users (adjusted HR = 1.40; 95% CI 0.77–2.55). Similarly, our sensitivity analysis showed no significant increased or decreased risk of acute kidney injury found among users of DPP4 inhibitors (Fig. 2). A significant association was found between DPP4 inhibitor initiation when compared to metformin initiation (adjusted HR = 1.85; 95% CI 1.10–3.12). When metformin was considered as a time-dependent variable, the association was no longer significant (adjusted HR = 1.39, 95% CI 0.91–2.11).

**Table 1:** Patient characteristics of new-users of DPP-4 inhibitors and sulfonylureas for each study cohort.

Characteristics	New-user cohort for Acute Kidney Injury		p-value	New-user cohort for Acute Respiratory Tract Infections		p-value	New-user cohort for Acute Pancreatitis		p-value
	DPP4i (n = 8,411)	SU (n = 28,990)		DPP4i (n = 6,290)	SU (n = 20,409)		DPP4i (n = 8.419)	SU (n = 28,858)	
Age in yrs (sd)	57.5 (12.2)	60.1 (13.7)	<0.01	57.1 (12)	59.1 (13.4)	<0.01	57.5 (12.2)	60.1 (13.7)	<0.01
Female	41.5%	41.3%	0.7	38.4%	37.7%	0.3	41.6%	41.3%	0.65
Measure of deprivation									
Least	9.4%	10.5%		9.9%	10.6%		9.5%	10.6%	
Most	11%	11.3%		10.9%	10.9%		11%	11.3%	
Unknown	46.7%	40.5%	<0.01	46.5%	40.8%	<0.01	46.6%	40.3%	<0.01
Diabetes duration, yrs (sd)	2 (1.8)	1 (1.5)	<0.01	2 (1.8)	1.1 (1.5)	<0.01	2 (1.8)	1 (1.5)	<0.01
Number of drugs in year prior to cohort entry									
0–4	8.9%	11%		10.8%	13.9%		8.9%	10.9%	
5–10	45.3%	41.7%		51%	48.7%		45.2%	41.6%	
11+	45.8%	47.3%	<0.01	38.2%	37.4%	<0.01	45.8%	47.5%	<0.01
HbA1c									
<6.5%	4.0%	6.3%		3.8%	5.7%		4.0%	6.4%	
6.5–7.5%	18.3%	15.4%		18.1%	14.7%		18.4%	15.5%	
7.5–9%	44.6%	32.2%		45%	32.4%		44.5%	32.3%	
9%+	32.6%	44.2%		32.6%	45.8%		32.6%	44%	
Unknown	<1.0%	1.9%	<*0.01*	<1.0%	1.3%	<*0.01*	<1.0%	1.8%	<0.01
e*GFR*<60	14.2%	19.5%	<0.01	12.9%	17.4%	<0.01	14.3%	19.9%	<0.01
Diagnoses in year prior to cohort entry									
Heart Failure	1.1%	1.7%	<*0.01*	<1.0%	1.1%	0.15	1.1%	1.8%	<0.01
Hypertension	18%	20.1%	<0.01	17.1%	19.2%	<0.01	17.9%	20.4%	<0.01
Cirrhosis	<1.0%	<1.0%	<*0.01*	<1.0%	<1.0%	*0.17*	<1.0%	<1.0%	<*0.01*
Dyslipidemia	3.7%	5.0%	<0.01	3.4%	4.7%	<0.01	3.6%	5.1%	<0.01
Peripheral vascular disease	<1.0%	<1.0%	<*0.01*	<1.0%	<1.0%	*0.03*	<1.0%	<1.0%	<*0.01*
Medications in year prior to cohort entry									
Metformin	93.2%	76%	<0.01	94.2%	79.4%	<0.01	93.2%	76.3%	<0.01
Acarbose	<1.0%	<1.0%	*S*	<1.0%	<1.0%	*S*	<1.0%	<1.0%	*S*
SGLT2 Inhibitors	<1.0%	<1.0%	<*0.01*	<1.0%	<1.0%	*0.016*	<1.0%	<1.0%	*0.01*
Meglitinide	<1.0%	<1.0%	<*0.01*	<1.0%	<1.0%	<*0.01*	<1.0%	<1.0%	<*0.01*
Thiazolidinedione	4.3%	1.9%	<0.01	4.4%	2.1%	<0.01	4.3%	1.9%	<0.01
Insulin	1.5%	1.5%	0.9	1.3%	1.4%	0.6	1.5%	1.5%	1

S = suppressed due to low number of events.

**Table 2:** Measures of frequency and association for acute outcomes of interest among new-users of DPP-4 Inhibitors (DPP4i) vs. sulfonylureas (SU), metformin, thiazolidinediones (TZD), or insulin.

	New-user cohort for Acute Kidney Injury		New-user cohort for Acute Respiratory Infections		New-user cohort for Acute Pancreatitis	
***COMPARATOR: SU***						
	DPP4i	SU	DPP4i	SU	DPP4i	SU
Number of patients	8411	28990	6290	20409	8419	28858
Person-years of follow-up, yrs	7539	23931	5187	15587	7561	23959
Number of Events	36	252	537	1694	7	34
Incidence per 1000 person-years (95%CI)	4.8 (3.5–6.6)	10.5 (9.3–11.9)	103.5 (95.1–112.7)	108.7 (103.6–114)	0.9 (0.5–1.9)	1.4 (1–2)
Crude HR (95%CI)	0.46 (0.32–0.65)	-ref-	0.94 (0.86–1.04)	-ref-	0.63 (0.28–1.43)	-ref-
Adjusted HR (95%CI)	0.81 (0.56–1.18)	-ref-	0.93 (0.84–1.04)	-ref-	1.03 (0.42–2.52)	-ref-
***COMPARATOR: METFORMIN***						
	DPP4i	Metformin	DPP4i	Metformin	DPP4i	Metformin
Number of patients	728	74249	470	53475	774	77817
Person-years of follow-up, yrs	546	64795	331	43006	7561	23959
Number of Events	16	365	36	4395	S	34
Incidence per 1000 person-years (95%CI)	29.3 (18.1–47.6)	5.6 (5.1–6.2)	108.7 (78.6–150.4)	102.2 (99.3–105.3)	S	1.4 (1–2)
Crude HR (95%CI)	5.17 (3.13–8.54)	-ref-	1.03 (0.74–1.43)	-ref-	S	-ref-
Adjusted HR (95%CI)	1.85 (1.10–3.12)	-ref-	0.91 (0.65–1.27)	-ref-	S	-ref-
***COMPARATOR: TZD***						
	DPP4i	TZD	DPP4i	TZD	DPP4i	TZD
Number of patients	13347	3347	9898	2563	13371	3347
Person-years of follow-up	12802	3617	8752	2517	12823	3630
Number of Events	63	17	891	245	12	S
Incidence per 1000 person-years (95%CI)	4.9 (3.9–6.3)	4.7 (2.9–7.5)	101.8 (95.3–108.7)	97.3 (85.9–110.3)	0.9 (0.5–1.6)	S
Crude HR (95%CI)	1.02 (0.59–1.74)	-ref-	1.01 (0.87–1.16)	-ref-	S	-ref-
Adjusted HR (95%CI)	1.12 (0.60–2.10)	-ref-	1.02 (0.86–1.21)	-ref-	1.21 (0.25–5.72)	-ref-
**COMPARATOR: INSULIN**						
	DPP4i	Insulin	DPP4i	Insulin	DPP4i	Insulin
Number of patients	13881	4918	10205	3286	13991	4884
Person-years of follow-up	13433	1693	9177	1085	13511	1710
Number of Events	64	26	922	161	14	5
Incidence per 1000 person-years (95%CI)	4.8 (3.7–6.1)	15.4 (10.5–22.5)	100.5 (94.2–107.2)	148.4 (127.2–173.2)	1 (0.6–1.7)	2.9 (1.3–6.8)
Crude HR (95%CI)	0.39 (0.24–0.63)	-ref-	0.72 (0.60–0.85)	-ref-	0.42 (0.15–1.22)	-ref-
Adjusted HR (95%CI)	1.40 (0.77–2.55)	-ref-	0.74 (0.60–0.92)	-ref-	1.01 (0.24–4.19)	-ref-

S = suppressed due to low number of events.

**Figure 3:**
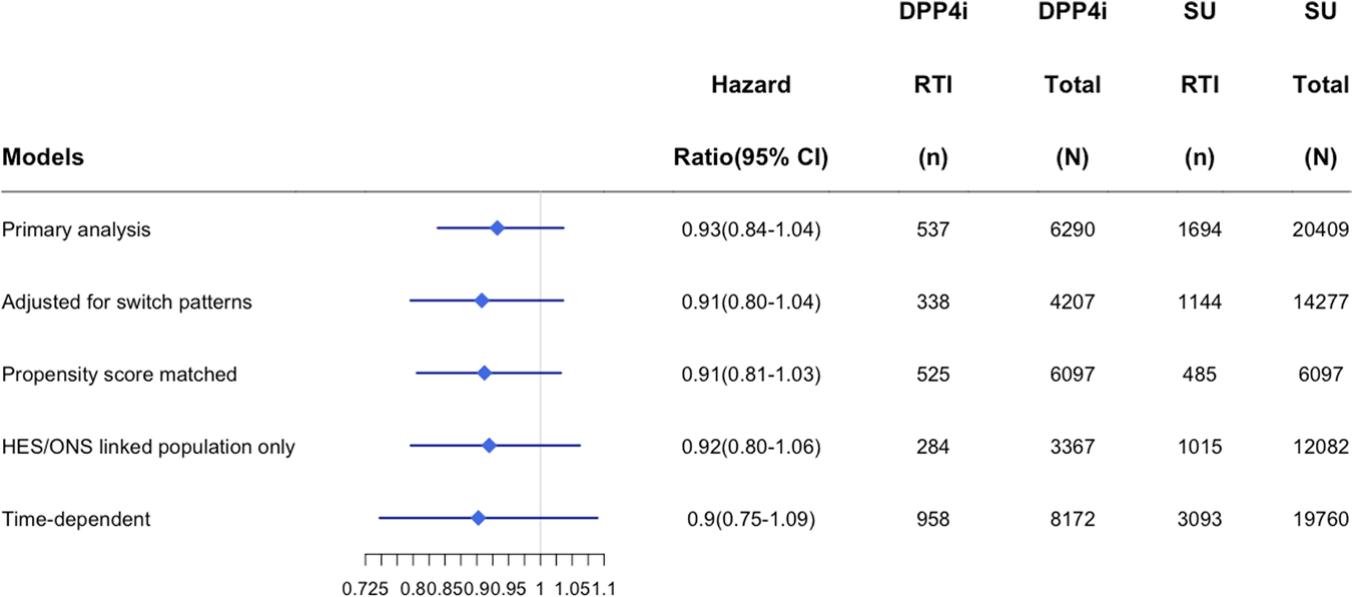
Sensitivity analyses for the association between DPP4 Inhibitors (DPP4i) and Sulfonylurea (SU) users for respiratory tract infection (RTI).

Within the acute respiratory tract infection cohort, there were 6,290 initiators of a DPP4 inhibitor and 20,409 initiators of a sulfonylurea (Fig. 1). Mean follow-up time was 284 days and ranged from 1 to 3023 days. Differences in populations prior to being matched by high-density propensity scores were similar to those in the acute kidney injury cohort (DPP4 users had longer duration of diabetes, fewer hospitalizations, fewer baseline HbA1C above 9%, and greater use of metformin in the past), and balanced out after matching (Table 2 and Supplementary Appendix Table S9). After 20,774 years of person-time follow-up this cohort experienced 2231 episodes of an incident acute respiratory tract infection among new users of DPP-4 inhibitors (n = 537, incidence rate 104 per 1000 person-years) and sulfonylureas (n = 1,694, incidence rate 109 per 1000 person-years) (Table 2). Both unadjusted and adjusted Cox proportional hazard models suggest DPP4 inhibitors are not associated with a significant increase or decrease in respiratory tract infection risk (HR = 0.94; 95% CI 0.86–1.04; adjusted HR = 0.93; 95% CI 0.84–1.04). Comparisons of DPP4 inhibitors with metformin (adjusted HR = 0.91, 95% CI 0.65–1.27) or thiazolidinediones (adjusted HR = 1.02; 95% CI 0.86–1.21) also did not demonstrate an increased risk; however, comparisons with insulin showed DPP4 inhibitors were associated with a decreased risk of an acute respiratory tract infection (adjusted HR = 0.74; 95% CI 0.60–0.92). In sensitivity analysis findings remained consistent with the primary analysis showing no significant increased or decreased risk of respiratory tract infections among users of DPP-4 inhibitors (Fig. 3). In a sub-group analysis, DPP4 inhibitors were not associated with an increased risk of an upper respiratory tract infection (adjusted HR = 0.94; 95% CI 0.84–1.05); however, when examining pneumonia and influenza specifically, DPP4 inhibitors were found to have a protective effect over sulfonylureas (adjusted HR = 0.63; 95% CI 0.44–0.90).

**Figure 4:**
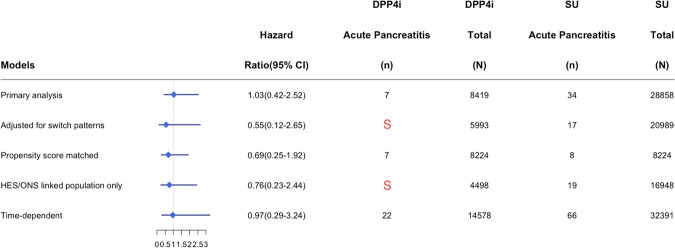
Sensitivity analyses for the association between DPP4 Inhibitors (DPP4i) and Sulfonylurea (SU) users for acute pancreatitis. S = suppressed due to low number of events.

Within the acute pancreatitis cohort, there were 8,419 initiators of a DPP4 inhibitor and 28,858 initiators of a sulfonylureas (Fig. 1). Mean follow-up time was 308 days and ranged from 1 to 3300 days. Differences in baseline characteristics among groups prior to being matched by high-density propensity scores were similar to those in the acute kidney injury and respiratory tract infection cohorts and balanced out after matching (Table 2). After 31,520 years of person-time follow-up this cohort experienced 41 episodes of acute pancreatitis among new users of DPP4 inhibitors (n = 7, incidence rate = 0.9 per 1000 person-years) and sulfonylureas (n = 34, incidence rate 1.4 per 1000 person-years) (Table 2). Neither crude nor adjusted hazard ratios demonstrate a significant difference (HR = 0.63; 95% CI 0.28–1.43; adjusted HR = 1.03; 95% CI 0.42–2.52). Comparisons with thiazolidinediones (adjusted HR = 1.21; 95% CI 0.25–5.72) and insulin (adjusted HR = 1.01; 95% CI 0.24–4.19) did not demonstrate an increased risk. There were too few events to compare DPP4 inhibitor initiators to metformin initiators; however, when DPP4 inhibitor use was not associated with an increased or decreased risk of acute pancreatitis when compared to metformin in a time-varying model (adjusted HR = 1.51; 95% CI 0.48–4.75). Due to a small number of events, we were limited in the number of sensitivity analyses that we could conduct on this relationship (Fig. 4).

## Discussion

Across a series of new-user cohorts consisting of patients with type 2 diabetes in the UK, we found that the incidence of acute kidney injury, respiratory tract infections, and acute pancreatitis were 4.8, 104, and 0.9 per 1,000 patient-years, respectively, among initiators of a DPP4 inhibitor. After adjusting for potential confounding factors, there was no statistically significant increased risk for either outcome when compared to new initiators of a sulfonylurea. These findings were robust and held in most secondary and sensitivity analyses.

Consistent with findings from randomized controlled trials and prior observational research^10,44–46^, our study suggests that DPP4 inhibitors do not increase the risk of acute kidney injury. Although our findings are comparable to observational studies evaluating the safety of saxagliptin^12^, two other observational studies have published inconsistent findings^10,11^. These discordant results may be potentially explained by chance or possible bias due to exposure definitions and time-related bias. For example, Shih *et al*., used the prescription termination date of DPP4 inhibitor to classify patients into current, recent, and past exposure categories; however, it is unclear if the current user category for which they observed the increased risk of an acute kidney injury was over-represented^10^. The risk of acute kidney injury was higher for current DPP4 inhibitor users, but not for recent or past users. The findings reported by Chao *et al*., appear to be susceptible to time-related bias as short-term DPP4 inhibitor users (<90 days duration) were excluded from the analysis, whereas no such exclusion criteria were applied to the control group^11^. Although we found an increased risk of acute kidney injury when comparing DPP4 inhibitor and metformin initiators, this comparison is highly susceptible to confounding by indication given metformin’s place in therapy and contraindication in patients with renal dysfunction. It is reasonable to suspect that there are unmeasured confounders at play that were considered during the prescribing process. Moreover, the substantial decrease in effect size between the crude (HR = 5.17) and adjusted (HR = 1.85) hazard ratios, as well as the lack of association within a time-dependent model suggest that a strong degree of confounding is present.

Prior observational studies examining the risk of DPP4 inhibitors and infections have examined all-cause infections^12^, upper respiratory tract infections^13^,or pneumonia^18,19,48^ as outcomes of interest. Similarly, the majority of meta-analysis pooling results from clinical trials suggest that there is not a significant risk of all-cause infection, upper respiratory tract infections, or pneumonia associated with using a DPP4 inhibitor. Our study expands on these findings using a contemporary follow-up period, multiple reference groups, and both upper and lower acute respiratory infections.

Several previous observational studies have examined the relationship between DPP4 inhibitors and acute pancreatitis. Our findings add to this evidence base suggesting that DPP4 inhibitors do not substantially increase the risk of acute pancreatitis compared to other glucose-lowering agents. However, given the small number of events, our study was limited in power to detect small or modest differences in the incidence of acute pancreatitis between exposure groups.

There are several limitations of this study to consider. As with any observational study, there is a possibility of residual and unmeasured confounding affecting study results. For example, the increased risk of acute kidney injury observed in our study for initiators of DPP4 inhibitors vs. metformin is susceptible to residual and unmeasured confounding given metformin’s contraindication in patients with renal impairment. We took steps to mitigate this risk, such as applying high-dimensional propensity scores to maximize the balance of measured baseline confounders. Second, our study cohorts all had limited follow-up time. Specifically, the mean follow-up times were 307, 284, and 308 days for the acute kidney injury, acute respiratory tract infection and acute pancreatitis cohorts, respectively. Therefore, our study is unable to quantify the long-term risks associated with DPP4 inhibitors. Third, our study, like others relying on secondary data sources, is susceptible to information bias via outcome measurement error. We used diagnostic codes that have been used previously, and which have variable positive predictive values (acute kidney injury ~17%; acute pancreatitis ~42%; respiratory tract infection ~97%)^49–51^. Finally, prescription data was used to measure exposure to diabetes therapies. It is possible that primary and secondary non-adherence may lead to an overestimation of exposure.

In conclusion, this study suggests that initiation of a DPP4 inhibitor was not associated with an increased risk of acute kidney injury, respiratory tract infections, or acute pancreatitis compared to sulfonylureas or other glucose-lowering therapies. Further studies are required to quantify the potential for within-class differences among DPP4 inhibitors, and to explore modifying factors with respect to the association between DPP4 inhibitors and acute kidney injury, acute respiratory tract infections, and acute pancreatitis.

## Electronic supplementary material

Supplemental Material
