# Supplementary material for: Comparative Safety of Dipeptidyl Peptidase-4 Inhibitors Versus Sulfonylureas and Other Glucose-lowering Therapies for Three Acute Outcomes
Source: Sci Rep. 2018 Oct 11;8:15142. doi: 10.1038/s41598-018-33483-y (PMC6181978; doi:10.1038/s41598-018-33483-y)
Supplement: Supplementary file 1 — Supplemental Material [file 41598_2018_33483_MOESM1_ESM.pdf]

Comparative Safety of Dipeptidyl Peptidase-4 Inhibitors Versus Sulfonylureas and Other Glucose-lowering Therapies for Three Acute Outcomes

John-Michael Gamble PhD[1,2], Eugene Chibrikov PhD[1,3], Jennifer R. Donnan MSc[2], Laurie K. Twells PhD[2,3], William K. Midodzi PhD[3], Sumit R. Majumdar MD, MPH[4].

[1]School of Pharmacy, Faculty of Science, University of Waterloo

[2]School of Pharmacy, Memorial University of Newfoundland

[2] Faculty of Medicine, Memorial University of Newfoundland

[3] Division of General Internal Medicine, Department of Medicine, University of Alberta

Corresponding author: John-Michael Gamble; email: jm.gamble@uwaterloo.ca

Supplemental Material

Table S1. Cohort Exclusion Criteria [medicines, diagnoses, and procedures assessed in the 365 days prior to study index date].

**ACUTE KIDNEY INJURY**	Diagnostic code in GOLD or HES for acute renal failure, dialysis or kidney transplant.<br>Procedure code for dialysis or kidney transplant.
**RESPIRATORY TRACT INFECTIONS**	Diagnostic code in GOLD or HES for malignancy, HIV infection, organ transplantation.<br>Prescription record for immunosuppressive medications, or ≥ 10 corticosteroids or antibiotics.
**ACUTE PANCREATITIS**	Diagnostic code in GOLD or HES for pancreatic cancer, pancreatic injury, defects of the pancrease, cystic fibrosis, or lupus.<br>Procedure code for pancreatectomy or bariatric surgery.

READ and ICD-10 Codes for Outcomes of Interest

Table S2: Read Codes for Acute Kidney Injury

READ Code	Description
K04..00	Acute Renal Failure
K040.00	Acute Renal Tubular Necrosis
K041.00	Acute Renal Cortical Necrosis
K042.00	Acute Renal Medullary Necrosis
K042.11	Necrotising Renal Papillitis
K043.00	Acute Drug-Induced Renal Failure
K044.00	Acute Renal Fail Urin Obstruct
K04y.00	Other Acute Renal Failure
K04z.00	Acute Renal Failure Nos
14V2.00	H/O: Renal Dialysis
14V2.11	H/O: Kidney Dialysis
7L1A.00	Compensation For Renal Failure
7L1A.11	Dialysis For Renal Failure
7L1A000	Renal Dialysis
7L1A011	Thomas Intravascular Shunt For Dialysis
7L1Ay00	Other Specified Compensation For Renal Failure
7L1Az00	Compensation For Renal Failure Nos
ZV45100	[V]Renal Dialysis Status
ZV56.00	[V]Aftercare Involving Intermittent Dialysis
ZV56000	[V]Aftercare Involving Extracorporeal Dialysis
ZV56011	[V]Aftercare Involving Renal Dialysis Nos
ZV56100	[V]Preparatory Care For Dialysis
ZV56y00	[V]Other Specified Aftercare Involving Intermittent Dialysis
ZV56y11	[V]Aftercare Involving Peritoneal Dialysis
ZV56z00	[V]Unspecified Aftercare Involving Intermittent Dialysis
ZVu3G00	[X]Other Dialysis
K0D..00	End-Stage Renal Disease
7L1A200	Haemodialysis Nec

7L1A300	Haemofiltration
7L1A700	Haemoperfusion
K06..00	Renal Failure Unspecified
K06..11	Uraemia Nos
K060.00	Renal Impairment
K060.11	Impaired Renal Function
Kyu2000	[X]Other Acute Renal Failure
A160200	Tuberculous Pyelonephritis
G500400	Acute Pericarditis - Uraemic
K100600	Calculous Pyelonephritis
K101.00	Acute Pyelonephritis
K101000	Acute Pyelonephritis Without Medullary Necrosis
K101100	Acute Pyelonephritis With Medullary Necrosis
K101z00	Acute Pyelonephritis Nos
K104.00	Xanthogranulomatous Pyelonephritis
K10y.00	Pyelonephritis And Pyonephrosis Unspecified
K10y000	Pyelonephritis Unspecified
K10y300	Pyelonephritis In Diseases Ec
K10yz00	Unspecified Pyelonephritis Nos
7L1A100	Peritoneal Dialysis
7L1A400	Automated Peritoneal Dialysis
7L1A500	Continuous Ambulatory Peritoneal Dialysis
7L1A600	Peritoneal Dialysis Nec
14S2.00	H/O: Kidney Recipient
7B00.00	Transplantation Of Kidney
7B00000	Autotransplant Of Kidney
7B00100	Transplantation Of Kidney From Live Donor
7B00111	Allotransplantation Of Kidney From Live Donor
7B00200	Transplantation Of Kidney From Cadaver
7B00211	Allotransplantation Of Kidney From Cadaver
7B00300	Allotransplantation Of Kidney From Cadaver, Heart-Beating
7B00400	Allotransplantation Kidney From Cadaver, Heart Non-Beating
7B00500	Allotransplantation Of Kidney From Cadaver Nec
7B00y00	Other Specified Transplantation Of Kidney
7B00z00	Transplantation Of Kidney Nos
ZV42000	[V]Kidney Transplanted

Table S3: ICD-10 Codes for Acute Kidney Injury

ICD-10	Description
N17.0	Acute renal failure with tubular necrosis
N17.1	Acute renal failure with acute cortical necrosis
N17.2	Acute renal failure with medullary necrosis
N17.8	Other acute renal failure
N17.9	Acute renal failure, unspecified

Table S4: Read Codes for Respiratory Infections

READ Code	Description
H0*	Acute respiratory infections
H12*	Chronic pharyngitis and nasopharyngitis
H13*	Chronic sinusitis
H14*	Chronic tonsil and adenoid disease
H15*	Peritonsillar abscess - quinsy
H16*	Chronic laryngitis and laryngotracheitis
H2*	Pneumonia and influenza

Table S5: ICD-10 Codes for Respiratory Infections

ICD-10 Code	Description
J00	Acute nasopharyngitis
J01	Acute sinusitis
J02	Acute pharyngitis
J03	Acute tonsillitis
J04	Acute laryngitis and tracheitis
J05	Acute obstructive laryngitis and epiglottitis
J06	Acute upper respiratory infections
J09	Influenza due to certain identified influenza virus
J10	Influenza due to other identified influenza virus
J11	Influenza, virus not identified
J12	Viral pneumonia, not elsewhere classified
J13	Pneumonia due to Streptococcus pneumonia
J14	Pneumonia due to Haemophilus influenza
J15	Bacterial pneumonia, not elsewhere classified
J16	Pneumonia due to other infectious organism, not elsewhere classified
J17	Pneumonia in diseases classified elsewhere
J18	Pneumonia, organism unspecified
J20	Acute bronchitis
J21	Acute bronchiolitis
J22	Unspecified acute lower respiratory infection

Table S6: Read Codes for Acute Pancreatitis

READ Code	Description
J670.	Acute Pancreatitis
J6700	Acute Pancreatitis unspecified
J6701	Acute recurrent pancreatitis
J6702	Acute Haemorrhagic pancreatitis
J6703	Acute Suppurative Pancreatitis
J6704	Subacute Pancreatitis
J6705	Gallstone Acute Pancreatitis
J6706	Idiopathic Acute Pancreatitis
J6707	Biliary Acute Pancreatitis
J6708	Alcohol-induced Acute Pancreatitis
J6709	Drug-induced Acute Pancreatitis
J670y	Other Acute Pancreatitis
J670z	Acute Pancreatitis Nos

Table S7: ICD-10 Codes for Acute Pancreatitis

ICD-10 Code	Description
K85.0	Idiopathic acute pancreatitis
K85.1	Biliary acute pancreatitis
K85.2	Alcohol-induced acute pancreatitis
K85.3	Drug-induced acute pancreatitis
K85.8	Other acute pancreatitis
K85.9	Acute pancreatitis, unspecified

Table S8: Covariates To Be Forced Into High-dimentional Propensity Score Models For Safety Events Of Interest [All covariates assessed in the 365 days prior to study index date]

Acute Kidney Injury	Respiratory Infections	Acute Pancreatitis
Age at index date	Age at index date	Age at index date
Alcohol Abuse [Never, Former, Current, Unknown]	Alcohol Abuse [Never, Former, Current, Unknown]	Alcohol Abuse [Never, Former, Current, Unknown]
BMI	BMI	BMI
Duration of treated diabetes [time between first oral antidiabetic drug and study index date]	Duration of treated diabetes [time between first oral antidiabetic drug and study index date]	Duration of treated diabetes [time between first oral antidiabetic drug and study index date]
History of:<br>Cirrhosis Congestive heart failure<br>Hypertension<br>Hyperlipidemia<br>Nephrolithiasis<br>Peripheral vascular disease	History of:<br>Cirrhosis Congestive heart failure<br>Hypertension<br>Hyperlipidemia<br>Ischemic heart disease<br>Peripheral vascular disease<br>Respiratory diseases	History of:<br>Cirrhosis Congestive heart failure<br>Hypertension<br>Hyperlipidemia<br>Nephrolithiasis<br>Peripheral vascular disease
Hospitalization	Hospitalization	Hospitalization
Most recent HbAlc value to index date	Most recent HbAlc value to index date	Most recent HbAlc value to index date
Number of distinct prescription drugs	Number of distinct prescription drugs	Number of distinct prescription drugs
Prior use of ACE-inhibitors, ARBs, NSAIDs, thiazides, loop diuretics, or statins	Number of GP visits for infections	Prior use of fibrates
Sex	Prior use of antibiotics, antivirals, influenza vaccine, pneumococcal vaccine, proton pump inhibitors, inhaled corticosteroid, inhaled anticholinergics or beta-agonists, or statins.	Sex
Smoking status [Never, Former, Current, Unknown]	Sex	Smoking status [Never, Former, Current, Unknown]
Socioeconomic status [Index of Mulitiple Deprivation]	Smoking status [Never, Former, Current, Unknown]	Socioeconomic status [Index of Mulitiple Deprivation]
Stages of chronic kidney disease [none, I, II, II, or IV]	Socioeconomic status [Index of Mulitiple Deprivation]	Use of other antidiabetic agentes [metformin, sulfonlyureas, TZDs, insulin, other]
Use of other antidiabetic agents [metformin, sulfonlyureas, TZDs, insulin, other]	Use of other antidiabetic agents [metformin, sulfonlyureas, TZDs, insulin, other]	Year of cohort entry
Year of cohort entry	Year of cohort entry	

Table S9: Patient Characteristics among DPP-4 and Sulfonylurea Initiators, matched 1:1 by Propensity Scores

	New-user cohort for Acute Kidney Injury		New-user cohort for Acute Respiratory Tract Infections		New-user cohort for Acute Pancreatitis	
**Characteristics**	DPP4i (n=8196)	SU (n=8196)	DPP4i (n=6097)	SU (n=6097)	DPP4i (n=8224)	SU (n=8224)
Age in yrs (sd)	57.6 (12.3)	57.5 (12.3)	57.1 (12)	57.4 (12.2)	57.6 (12.3)	57.6(12.4)
Female	41.4%	41.5%	38.3%	39.3%	41.7%	41.8%
Measure of deprivation						
Least	9.4%	9.6%	9.9%	9.8%	9.6%	9.5%
Most	11%	10.9%	10.9%	11.4%	11%	11.2%
Unknown	46.4%	46.3%	46.2%	45.7%	46.4%	46.0%
Diabetes duration, yrs (sd)	1.9 (1.7)	1.9 (1.8)	1.9 (1.7)	1.9 (1.8)	1.9 (1.7)	1.9 (1.9)
Number of drugs in year prior to cohort entry						
0-4	9.0%	8.7%	10.9%	10.3%	8.9%	8.6%
5-10	44.9%	44.1%	50.9%	50.1%	44.9%	45.0%
11+	46.1%	47.2%	38.2%	39.6%	46.2%	46.4%
HbA1c						
<6.5%	4.1%	3.8%	3.8%	3.8%	4.1%	4.1%
6.5-7.5%	18.1%	17.3%	17.9%	17.4%	18.0%	17.4%
7.5-9%	44.1%	44.0%	44.4%	43.9%	44.1%	43.8%
9%+	33.2%	34.4%	33.4%	34.5%	33.2%	34.3%
Unknown	<1%	<1%	<1%	<1%	<1%	<1%
e*GFR* <60	14.2%	14.1%	12.9%	13.8%	14.4%	14.4%
Diagnoses in year prior to cohort entry						
**Heart Failure**	1.1%	1.0%	<1%	<1%	1.1%	<1%
**Hypertension**	18.1%	18.2%	17.3%	17.7%	18.0%	17.8%
**Cirrhosis**	<1%	<1%	<1%	<1%	<1%	<1%
**Dyslipidemia**	3.7%	3.8%	3.4%	3.6%	3.7%	3.8%
**Peripheral vascular disease**	<1%	<1%	<1%	<1%	<1%	<1%
Medications in year prior to cohort entry						
**Metformin**	93.1%	94.2%	94.0%	95.2%	93.0%	93.9%
**Acarbose**	<1%	<1%	<1%	<1%	<1%	<1%
**SGLT2 Inhibitors**	<1%	<1%	<1%	<1%	<1%	<1%
**Meglitinide**	<1%	<1%	<1%	<1%	<1%	<1%
**Thiazolidinedione**	3.7%	3.4%	3.9%	3.5%	3.9%	3.4%
**Insulin**	1.5%	1.4%	1.3%	1.5%	1.5%	1.4%
